# Dental Implants in Patients with Oral Lichen Planus: A Systematic Review

**DOI:** 10.3390/medicina56020053

**Published:** 2020-01-27

**Authors:** Bruno Ramos Chrcanovic, Aline Fernanda Cruz, Ricardo Trindade, Ricardo Santiago Gomez

**Affiliations:** 1 Department of Prosthodontics, Faculty of Odontology, Malmö University, 214 21 Malmö, Sweden; 2 Department of Oral Surgery and Pathology, School of Dentistry, Universidade Federal de Minas Gerais, Belo Horizonte 31270-901, Brazil; alinecruzz16@gmail.com (A.F.C.); rsgomez@ufmg.br (R.S.G.); 3 Department of Prosthodontics, Faculty of Odontology, The Sahlgrenska Academy, University of Gothenburg, 405 30 Gothenburg, Sweden; ricardo.bretes.trindade@gu.se

**Keywords:** oral lichen planus, dental implants, osseointegration, failure, recommendations

## Abstract

*Background and Objectives*: To integrate the available published data on patients with oral lichen planus (OLP) rehabilitated with dental implants, as well as to review the recommendations for OLP patients receiving implants. *Materials and Methods*: An electronic search was undertaken in February 2019 using five databases. Publications reporting cases of patients with OLP and rehabilitated with implant-supported oral prosthesis were included. *Results*: Twenty-two publications were included (230 patients, 615 implants). The overall implant failure rate was 13.9% (85/610). In patients with oral squamous cell carcinoma (OSCC) the failure rate was 90.6% (29/32), but none of these implants lost osseointegration; instead, the implants were removed together with the tumor. One study presented a very high implant failure rate, 76.4% (42/55), in patients with “active lichen planus”, with all implants failing between 7–16 weeks after implant placement, and its conflicting and incongruent results are discussed in detail. There was a statistically significant difference between the failure rates in implants installed in different jaws (maxilla/mandible) and when implants of different surfaces were used (turned/moderately rough), but not between patients with reticular or erosive OLP types, or between male and female patients. If OSCC patients and the cases of the latter study are not considered, then the failure rate becomes very low (2.7%, 14/523). The time between implant placement and failure was 25.4 ± 32.6 months (range 1–112). The mean ± SD follow-up was 58.9 ± 26.7 months (1–180). *Conclusions*: When the results of the one study with a very high failure rate and of the cases that developed OSCC are not considered, the dental implant failure rate in OLP patients was 2.7% after a follow-up of approximately five years. Recommendations are given when treating OLP patients with dental implants.

## 1. Introduction

Oral lichen planus (OLP) is an autoimmune and chronic inflammatory disease affecting the oral mucosa [1]. The precise etiology of OLP is still unknown, but it is believed to be associated with a cell-mediated immune dysregulation caused by the interaction between genetic and environmental factors [2]. The prevalence of OLP in the general population is around 2.0%, with a significantly higher prevalence in women [3]. The oral lesions can occur in six distinct clinical forms, of which some can cause erosion of the mucosal epithelium with subsequent burning sensation and sometimes pain.

Mucosal diseases such as OLP have been suggested to negatively affect the ability of the epithelium to attach to titanium surfaces. Hence, it has been postulated that, when compared to healthy peri-implant mucosa, the peri-implant mucosa affected by OLP may respond differently to a bacterial challenge, resulting in a faster breakdown of the peri-implant soft tissue seal [4]. On account of this, the eligibility of OLP patients to receive dental implants has been questioned. The aim of the present review was to integrate the available data published in the literature on patients with OLP rehabilitated with dental implants, as well as to review the recommendations for OLP patients receiving implants.

## 2. Materials and Methods

This study followed the PRISMA Statement guidelines [5].

### 2.1. Objective

The purpose of the present study was to integrate the available data published in the literature on patients with OLP rehabilitated with dental implants. The focused question was elaborated by using the PICO format (participants, interventions, comparisons, outcomes): What is the failure rate of dental implants used for oral rehabilitation in patients with OLP? Furthermore, the objective was to review the recommendations for OLP patients receiving implants.

### 2.2. Search Strategies

An electronic search without time restrictions was undertaken in February 2019 using the following databases: PubMed/Medline, Web of Science, Science Direct, J-Stage, and Lilacs. The following terms were used in the search strategies: (oral lichen planus) AND (dental implant OR oral implant).

Google Scholar was also checked. A manual search of dental implants-related journals, including British Journal of Oral and Maxillofacial Surgery, Clinical Implant Dentistry and Related Research, Clinical Oral Implants Research, Implant Dentistry, International Journal of Oral Implantology (formerly European Journal of Oral Implantology), International Journal of Oral and Maxillofacial Implants, International Journal of Oral and Maxillofacial Surgery, International Journal of Periodontics and Restorative Dentistry, International Journal of Prosthodontics, Journal of Clinical Periodontology, Journal of Dental Research, Journal of Craniofacial Surgery, Journal of Cranio-Maxillofacial Surgery, Journal of Maxillofacial and Oral Surgery, Journal of Oral Implantology, Journal of Oral and Maxillofacial Surgery, Journal of Oral Rehabilitation, Journal of Periodontology, Oral Surgery Oral Medicine Oral Pathology Oral Radiology and Endodontology, and Quintessence International, was performed. The reference list of the identified studies and the relevant reviews on the subject were also checked for possible additional studies. 

### 2.3. Inclusion and Exclusion Criteria

Eligibility criteria included clinical human studies, either randomized or not, reporting cases of patients with OLP and rehabilitated with implant-retained and/or implant-supported oral prosthesis. There were no time or language restrictions for the publications. Exclusion criteria were technical reports, animal studies, in vitro studies, and review papers.

### 2.4. Study selection

The titles and abstracts of all reports identified through the electronic searches were read independently by the authors. For studies appearing to meet the inclusion criteria, or for which there were insufficient data in the title and abstract to make a clear decision, the full report was obtained. Disagreements were resolved by discussion between the authors. 

### 2.5. Data extraction

The review authors independently extracted data using specially designed data extraction forms. Any disagreements were resolved by discussion. For each of the identified studies included, the following data were then extracted: the patient’s sex and age, OLP clinical form, implant location (maxilla/mandible), implant healing time, implant surface, implant failure, time to failure, and follow-up period. Contact with authors for possible missing data was performed.

### 2.6. Analyses

A descriptive analysis was performed based on mean, standard deviation (SD), and percentage values. Comparisons of implant survival between some factors were done using the log-rank test. The degree of statistical significance was considered *p* < 0.05. All data were analyzed using IBM SPSS Statistics for Windows, version 25.0 (IBM Corp., Armonk, NY, USA). 

## 3. Results

### 3.1. Literature Search

**Figure 1:**
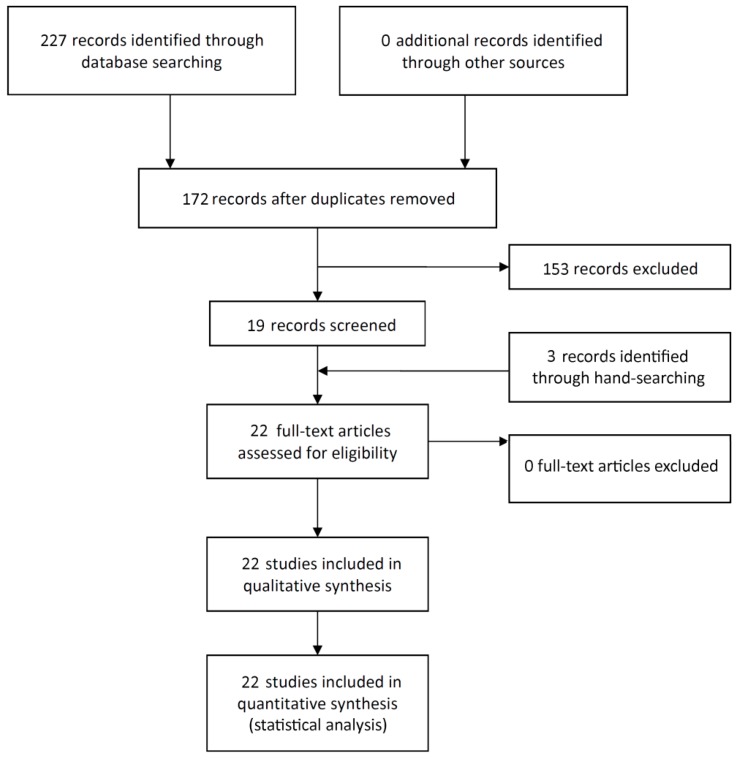
Study screening process.

The study selection process is summarized in Figure 1. The search strategy in the databases resulted in 227 papers, three papers through hand-searching, and no additional eligible papers were found in Google Scholar. In the end, a total of 22 publications were included.

### 3.2. Description of the Studies and Analyses

The 22 included publications [6,7,8,9,10,11,12,13,14,15,16,17,18,19,20,21,22,23,24,25,26,27] reporting on 230 patients with OLP and rehabilitated with 615 dental implants (Table 1). There were 158 women (68.7%) and 72 men (31.3%), with a female:male ratio of 2.19:1. There was no information on implant survival for one patient with five implants. The global implant failure rate was 13.9% (85/610). If only the patients (*n* = 10) who developed oral squamous cell carcinoma (OSCC) were considered, then the failure rate increased to 90.6% (29/32). However, none of the implants in OSCC patients lost osseointegration; the implants were removed because they were in the area of the malignant tumor. One study [6] in particular presented a very high implant failure rate of 76.4% (42/55), in patients with “active lichen planus”, with all implants failing between 7 and 16 weeks after implant placement. If OSCC patients and the cases of Aboushelib et al. [6] are not considered, then the failure rate becomes very low (2.7%, 14/536). There was available information on the time of failure for 12 of these 14 failures, a mean ± SD of 25.4 ± 32.6 months (range, 1–112). There was information on the OLP clinical subtype for patients rehabilitated with 255 implants, comprising 162 implants in patients with the erosive form and 93 implants in patients with the reticular form. Patients presenting the reticular type presented a higher failure rate in comparison to patients with the erosive type, but the difference was not statistically significant (24.7% vs. 17.2%, respectively; *p* = 0.273, log-rank test). The difference in the failure rate between turned/machined and moderately rough implants was statistically significant (*p* < 0.001, log-rank test), and between implants installed in the maxilla in comparison to the implants installed in the mandible (*p* = 0.022, log-rank test), but not when implants in males and females were compared (*p* = 0.890, log-rank test). Most of the studies did not provide information separated by patient of whether the implant failures occurred in patients with one OLP clinical form or the other. The patients were followed up for a mean ± SD of 58.9 ± 26.7 months (range 1–180).

**Figure 2:**
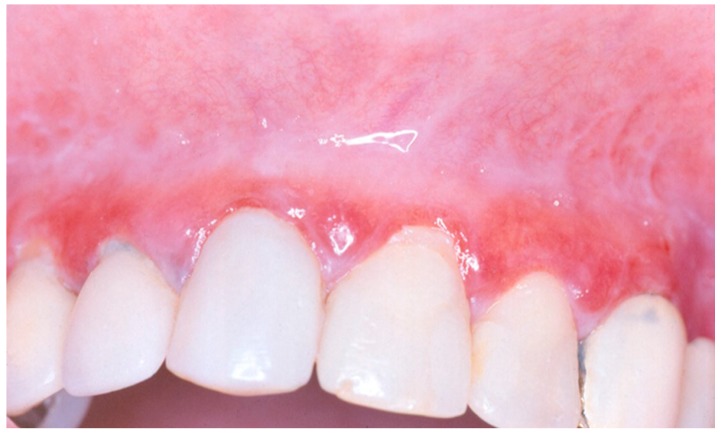
Typical clinical presentation of OLP erosive type, on the gingiva of maxillary anterior teeth.

**Figure 3:**
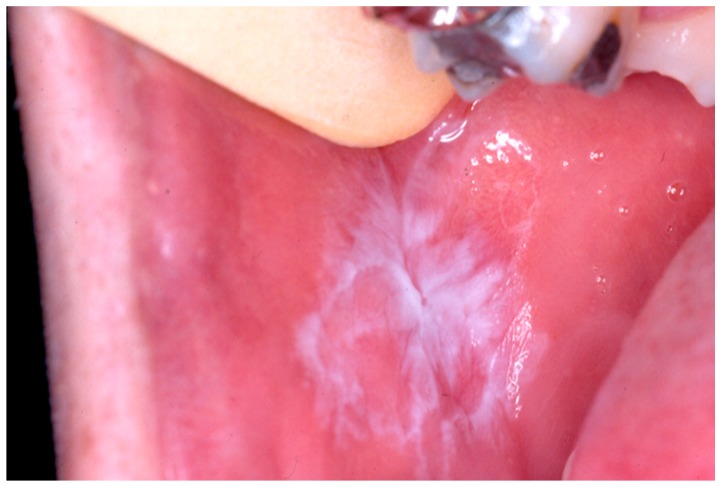
OLP with clinical presentation predominantly reticular type, on the buccal mucosa.

Figure 2 shows an example of the typical clinical presentation of an OLP erosive type, and Figure 3 of an OLP reticular type.

## 4. Discussion

The use of implant therapy requires consideration of the potential benefits of the therapy. To better appreciate this potential, the present study aimed to integrate the available data published in the literature on patients with OLP rehabilitated with dental implants. A review of rare clinical cases and conditions provides valuable information that allows professionals to make improved decisions and refine treatment plans to optimize clinical outcomes [28,29,30,31].

The authors of the present review consider that the implant failure rate in patients with OLP is very low (2.7%), after a mean follow-up time of approximately five years. This low rate occurred after not considering the data from a study that presented an implant failure rate of 76.4% [6], and also by excluding the cases of patients that developed OSCC. The disagreements of the results of this [6] and another study of the same group [19] with many other results from the literature (see the following paragraphs) could be a matter of chance, but the presence of so many incongruences, confusing reports, and some conflicting data makes one think over these results. Is the simple manifestation of OLP strong enough to overcome all other confounding factors and play such a powerful negative influence on osseointegration? If the results of all studies included in the present review are taken into consideration, one can say that the answer is not entirely clear yet. However, since the results of these two studies [6,19] just do not add up, one may suggest that the implant failure rate in patients with OLP is fairly low. Therefore, it is important to make some comments and explain the reasons why the results of Aboushelib et al. [6] were not considered here.

Such extremely high implant failure rate observed by Aboushelib et al. [6] is not expected even in patients irradiated in the head and neck region [32] or for implants placed in sites with the worst bone quality [33]. Despite quoting the first study twice, the second publication [19] made no comment or discussion about the alarming results found in the first one [6]. It was stated that the 23 patients of the study were “suffering from active lichen planus”, but it was not informed whether or not the patients were under some therapy for OLP, being corticosteroid or any other. Neither was it explained which clinical form of OLP (erosive, reticular, etc.) the patients presented and if they presented repeated episodes. These 42 lost implants were reimplanted after only one month and all these 42 reimplantations survived after a follow-up of 3 years. This goes against the results of several publications that observed that dental implants replacing failed implants had much lower survival rates than the rates reported for the previous attempts of implant placement [34,35,36,37,38]. Despite the condition having an established prevalence in females, Aboushelib et al. [6] and Khamis et al. [19] reported that nearly half of their patients were males. Furthermore, the authors of the first study failed to include two important controls: a healthy control versus the OLP study group, and after the implant removal a protocol healing group (corticosteroids and laser) versus a natural healing control group. Without the aforementioned controls, it is not possible to conclude whether the implant loss was caused by other factors unrelated to OLP.

In a response to a letter to the editor concerning the article of 2017 [6], Aboushelib [39] stated that “after insertion of the implant, accumulation of inflammatory cell infiltration prevents normal healing of the mucosa and starts to initiate both immediate and delayed allergic reactions, ending in (sic) attachment to the implant/bone interface, thus preventing successful osteointegration.” No references to support such statements were quoted. There are some considerations to be made concerning this statement. First, the reaction present in OLP is not of an allergic type, but of an autoimmune type [40]. Second, in order for this to happen, the lesion would have to be affecting the surgical site of all implants. This information was, however, not available neither from the first study [6] nor from the second [19]. And further to this important last point, the reason for implant failure is assumed to be from a soft tissue failure; however, osseointegration is a process of the bone as a tissue. Loss of implants due to factors initially affecting the mucosa and leading to peri-implantitis is a long-term event and therefore, would not justify the results found by Aboushelib et al. [6], since all implants were lost within 7 to 16 weeks, and some part of them were still submerged. A study comparing the prevalence of peri-implant mucositis in patients with OLP and healthy patients did not show an association between the presence of the disease and the increased prevalence of peri-implant mucositis [17]. These data were confirmed by a cross-sectional study that made the same comparison between the groups and also found no significant differences [20]. Interestingly, patients diagnosed with OLP who had desquamative gingivitis (DG) presented an increased prevalence of peri-implant mucositis; however, local factors such as the plaque index, stomatological condition per se, or both could be associated with the mucositis [17].

The authors [6] were very careful in reporting each patient’s sex, age implant location, socket condition, and loading technique in a table, but did not present the immunological results separated by patient, or which patients lost implants and the CD8 cell count for each of these patients. Neither mean values nor standard deviation was reported. The authors mentioned in the discussion (not in the results) that “appeared to be a direct correlation (*r* = 0.87) between the accumulation of immune mediators at the mucosa/soft tissue interface and normal wound healing around dental implants, resulting in interruption of soft tissue healing and successful osseointegration process.” As the only component reported to present high values was CD8, it is assumed that the authors directly linked the high count of this cell with the lack of osseointegration and the high implant failure rates. In a recent in vivo experimental biomaterials study, it was found that the gene expression of CD4 was increased in both the material that osseointegrated (titanium) and the materials that, for different reasons, did not osseointegrate. On the other hand, all these materials also had downregulated CD8 expression, which indicates that T cells are important for biomaterial bone integration, but the mechanisms are still not clear in terms of successful or failed osseointegration [41]. Aboushelib et al. [6] did not test any of many other factors that theoretically could have had an impact on the osseointegration (see next paragraph) and their statements concerning CD8 are still not clearly proven.

From the biochemical point of view of peri-prosthetic osteolysis, some inflammatory mediators have already been identified as pro-inflammatory actors (such as the cytokines IL-1, IL-6, IL-8, prostaglandin E2 (PGE2), RANKL, M-CSF, tumor necrosis factor-alpha) or anti-inflammatory (IL-10 and IL-13) [42,43]. In OLP, due to the immunoinflammatory nature of the disease [44], a local increase in cytokine levels and changes in the expression of molecules responsible for cell adhesion can occur [45]. Interestingly, some of these cytokines present altered expression in both processes approached in this work. In 2017, a meta-analysis showed that, in patients with OLP, IL-10 expression is reduced in serum and saliva [46]. IL-10 is an anti-inflammatory cytokine that can suppress bone resorption by osteoclastic suppression and regulation of bone homeostasis [47]. TNF-α is another cytokine that plays an important role in the regulation of inflammatory response in many autoimmune diseases by T cells activation and apoptosis induction [48]. The levels of this cytokine were significantly increased in the serum of patients with OLP when compared to healthy control [49]. Considering that increased TNF-α expression can be considered as a predictor of implant complications (such as bone dehiscence at the implant surface, implant rotational instability, implant laxity, and implant removal) [50], it would be interesting to investigate the difference of its levels in patients who received implant with and without OLP and its association with the clinical outcome of the implant. Understanding the events that disrupt the immune balance in peri-prosthetic osteolysis after implant placement is still a challenge. Additional molecular biology studies are necessary to elucidate the role of these and other biomarkers in osseointegration of implants in patients diagnosed with OLP. Given that all these factors could have a significant negative effect on the osseointegration process, one can only hypothesize, as no study has ever tested the relationship of these factors in OLP patients in relation to dental implants.

### 4.1. Recommendations

As the implant failure rate is assumed to be low in OLP patients, being comparable to that of patients with healthy oral mucosa, the rehabilitation of OLP patients with implant-supported prostheses would avoid the compression and irritation of the oral mucosa [8]. It is recommended that implant installation surgery be carried out in phases of remission of OLP [11,17,26]. Attention should also be given to the DG related to OLP [17], as the presence of DG has been associated with a higher rate of peri-implant mucositis in implants placed in OLP patients, although no correlation between DG and increased marginal bone loss (MBL) could be detected [17]. Even though MBL in patients with OLP is comparable to the bony resorption in healthy patients [11,17], meticulous oral hygiene and regular follow-ups are important not only to control the possibility of peri-implant mucositis and peri-implantitis, as in any patient with dental implants, but also for the early detection of malignant transformation into OSCC in the vicinity of dental implants [22]. This is very important, as peri-implantitis may closely resemble OSCC [7,10,12,16,21,22,25], with clinical signs such as gingival overgrowth, bone loss, and a tendency to bleed, although there is no epidemiologic evidence that dental implants pose any specific risk for cancer [25]. Delays in the diagnosis of peri-implantitis may pose negative consequences for the implant [51,52,53], but delayed diagnoses of malignancies may have dire implications for patient survival [25].

### 4.2. Limitations

The limitations of the present review include the fact that some studies were retrospective, which inherently results in errors, with incomplete records. Secondly, most of the case series did not provide data separately for each included patient, which would improve the quality of the statistical analyses [54]. Thirdly, many of the published cases had a short follow-up. The report of more cases followed up for many years would help us make a more reliable prognosis for the oral rehabilitation of OLP patients with dental implants in the long term.

## 5. Conclusions

The dental implant failure rate in OLP patients was 2.7% after a follow-up of approximately five years. When OLP patients developed oral squamous cell carcinoma (OSCC) the implant failure rate was 90.6%, but none of these implants lost osseointegration; the implants were removed together with the tumor. There are some recommendations to take into consideration when patients presenting OLP are intended to be rehabilitated with dental implants.

## Figures and Tables

**Table 1:** Demographic and clinical features of patients with oral lichen planus rehabilitated with dental implants described in the literature.

Variables	
**Patients** (*n*)	230
Male (%)/Female (%)	72 (31.3)/158 (68.7)
All studies, except Aboushelib et al. (2017)	41 (24.8)/124 (75.2)
Aboushelib et al. (2017)	31 (47.7)/34 (52.3)
Age (years), mean ± SD (min–max)	57.0 ± 10.7 (20–87)
**Implants** (*n*)	615
**Implant Failure** (%) ^a^	
Global	85/610 (13.9)
OSCC patients ^b^	29/32 (90.6)
Aboushelib et al. (2017)	42/55 (76.4)
Time between implant placement and failure ^c^, (months), mean ± SD (min–max)	1.9 ± 0.4 (1.6–3.7; *n* = 42)
Not including Aboushelib et al. (2017) or OSCC patients	14/523 (2.7)
Time between implant placement and failure ^d^, (months), mean ± SD (min–max)	25.4 ± 32.6 (1–112; *n* = 12)
Maxilla ^e^	34/195 (17.4)
Mandible ^e^	50/313 (16.0)
Male ^e^	21/170 (12.4)
Female ^e^	64/440 (14.5)
OLP erosive type ^e^	27/157 (17.2)
OLP reticular type ^e^	23/93 (24.7)
Turned implants ^e^	5/6 (83.3)
Moderately rough implants ^e^	43/247 (17.4)
**Follow-up time ^f^ (months), mean ± SD (min–max)**	58.9 ± 26.7 (1–180; *n* = 591)

SD – standard deviation, OSCC – oral squamous cell carcinoma, OLP - oral lichen planus; ^a^ There was no information on survival for 1 patient (5 implants); ^b^ These implants did not lose osseointegration, but were removed together with the OSCC; ^c^ Only the cases of Aboushelib et al. (2017); ^d^ Not including Aboushelib et al. (2017) or OSCC patients; there was no precise information about failure time for 2 implants; ^e^ For the cases with available information; ^f^ There was no precise information about follow-up time for 24 implants.
